# Functional investigation and two-sample Mendelian randomization study of neuropathic pain hub genes obtained by WGCNA analysis

**DOI:** 10.3389/fnins.2023.1134330

**Published:** 2023-04-14

**Authors:** Jianfeng Zeng, Cong Lai, Jianwei Luo, Li Li

**Affiliations:** ^1^Department of Anesthesiology, Sun Yat-sen Memorial Hospital, Sun Yat-sen University, Guangzhou, China; ^2^Department of Urology, Sun Yat-sen Memorial Hospital, Sun Yat-sen University, Guangzhou, China

**Keywords:** neuropathic pain, weighted gene co-expression network analysis, hub genes, IL2, Mendelian randomization

## Abstract

**Objective:**

Neuropathic pain as a complex chronic disease that occurs after neurological injury, however the underlying mechanisms are not clarified in detail, hence therapeutic options are limited. The purpose of this study was to explore potential hub genes for neuropathic pain and evaluate the clinical application of these genes in predicting neuropathic pain.

**Methods:**

Differentially expressed analysis and weighted gene co-expression network analysis (WGCNA) was used to explore new neuropathic pain susceptibility modules and hub genes. KEGG and GO analyses was utilized to explore the potential role of these hub genes. Nomogram model and ROC curves was established to evaluate the diagnostic efficacy of hub genes. Additionally, the correlation of IL-2 with immune infiltration was explored. Finally, a Mendelian randomization study was conducted to determine the causal effect of IL-2 on neuropathic pain based on genome-wide association studies.

**Results:**

WGCNA was performed to establish the networks of gene co-expression, screen for the most relevant module, and screen for 440 overlapping WGCNA-derived key genes. GO and KEGG pathway enrichment analyses demonstrated that the key genes were correlated with cytokine receptor binding, chemokine receptor binding, positive regulation of JAK–STAT cascade, chemokine-mediated signaling pathway, PI3K-AKT pathway and chemokine pathway. Through Cytoscape software, top ten up-regulated genes with high scores were IL2, SMELL, CCL4, CCR3, CXCL1, CCR1, HGF, CXCL2, GATA3, and CRP. In addition, nomogram model performed well in predicting neuropathic pain risk, and with the ROC curve, the model was showed to be effective in diagnosis. Finally, IL2 was selected and we observed that IL2 was causally associated with immune cell infiltrates in trigeminal neuralgia. In inverse variance weighting, we found that IL2 was associated with the risk of trigeminal neuralgia with an OR of 1.203 (95% CI = 1.004–1.443, *p* = 0.045).

**Conclusion:**

We constructed a WGCNA-based co-expression network and identified neuropathic pain-related hub genes, which may offer further insight into pre-symptomatic diagnostic approaches and may be useful for the study of molecular mechanisms for understanding neuropathic pain risk genes.

## 1. Introduction

Neuropathic pain is defined as chronic pain directly or indirectly caused by a pathological disorder or disease of the peripheral or central somatosensory nervous system (Finnerup et al., 2016). Unlike acute pain, which alerts and “protects” the body, neuropathic pain can persist without any benefit to the body and even damage it, seriously affecting the patient’s quality of life (St, 2018). The neuropathic pain is divided into central neuropathic pain and peripheral neuropathic pain depending on the site of injury. Neuropathic pain the most common type of chronic pain, has become a commonly seen chronic condition in many countries. The patients’ quality of life is seriously affected by neuropathic pain, which brings a heavy economic burden to society and individuals (Moisset and Page, 2021). Its pathogenesis is complex and no effective therapeutic drugs are available. Recently, some researches have combined bioinformatics approaches to elucidate the mechanisms of oxidative stress, neuroinflammation, ion channel alterations, activation of immune cell, glial-derived mediators and epigenetic modulation in neuropathic pain (Finnerup et al., 2021; Golubovic et al., 2021; Seo and Jun, 2021). Nevertheless, none of the various studies detected hub genes related to neuropathic pain by weighted gene co-expression network analysis (WGCNA). Thus, for gaining insight into the molecular mechanisms of neuropathic pain, it is essential to combine bioinformatics approaches to explore neuropathic pain-associated biomarkers.

It is essential to identify expression of specific genes in disorders to understand the microscopic mechanisms of many diseases such as neuropathic pain and to identify biomarkers relevant to diagnosis and therapeutic evaluation (Barclay et al., 2007; von Schack et al., 2011). In fact, several bioinformatics software and databases have been established to recognize disease-associated pathways, such as WGCNA, Kyoto Encyclopedia of Genes and Genomes (KEGG) enrichment analysis, and gene set enrichment analysis (Nangraj et al., 2020). WGCNA has also been applied to a wide variety of data, including proteomics data, genetic marker data, gene expression profiles, metabolomics information, and other high-dimensional data (Nangraj et al., 2020). Furthermore, WGCNA is useful for screening therapeutic targets or candidate biomarkers. Therefore, the current study was conducted to find related genes, new biomarkers, or potential mechanisms related to neuropathic pain.

Mendelian randomization (MR) is a reliable method that has been promoted in recent years to infer potential causal relationships, using single nucleotide polymorphisms (SNPs) as instrumental variables (IVs) to assess the causal relationship between exposure factors and outcomes (Jansen et al., 2014; Emdin et al., 2017). MR uses genetic variations strongly associated with exposure factors as instrumental variables to infer causal effects between exposure factors and study outcomes.

In this work, we identified differentially expressed genes (DEGs) through exploring data from the rattus norvegicus spinal nerve ligation (SNL) and sham groups. WGCNA was then utilized to recognize the most relevant modules for neuropathic pain, which greatly narrowing the scope of genes to screen for. In brief, we identified four hub genes, namely IL2, CCR3, CXCL1, CCL4, and SELL, as potential diagnostic markers that may be useful for the diagnosis of neuropathic pain and further understanding of the underlying mechanisms of neuropathic pain risk genes. Finally, the causal relationship between IL2 and neuropathic pain was explored through MR study.

## 2. Materials and methods

### 2.1. Data source

This dataset was created through transcriptome microarray assays in the Dorsal Root Ganglion (DRG) obtained from rattus norvegicus that underwent SNL (*N* = 10) or sham groups (*N* = 10). The expression data of all measured genes can be downloaded from the Gene Expression Omnibus (GEO) database.^1^

### 2.2. Differentially expressed genes identification

First, we read the data of dataset GSE24982 using *R* software (version 3.6.1) and preprocessed it for batch correction and normalization. Then we performed DEG analysis screening using the “limma” package. After significance analysis of expression levels, the “pheatmap” and “ggplot2” *R* packages were processed to generate volcano maps and DEG expression heat maps.

### 2.3. Weighted gene co-expression network analysis

WGCNA, a systematic approach to biology, is often applied to characterize patterns of genetic association between different samples. It is used for identifying highly synergistic genomes. Based on the interrelatedness of genomes and the relationship of genomes with phenotypes, it can be applied to identify candidate markers (Langfelder and Horvath, 2008). We constructed a gene co-expression network for neuropathic pain using the “WGCNA” *R* package. Finally, we evaluated the correlation of different modules with the pathogenic mechanism of neuropathic pain and selected the most relevant module as the central gene derived from WGCNA.

### 2.4. Screening of candidate pivotal genes and Go/KEGG analysis

These intersecting genes are regarded as candidate hub genes relevant for neuropathic pain pathogenesis. KEGG serves as a database resource for systematically analyzing gene function (Kanehisa and Goto, 2000). Subsequently, we performed Gene Ontology (GO) and KEGG enrichment analysis using the “clusterProfiler” *R* package in order to assist us in understanding the potential mechanisms of progression and pathogenesis.

### 2.5. Protein–protein interaction network hub genes

We predicted and visualized molecular interaction and protein–protein interaction (PPI) networks using STRING^2^ and Cytoscape software^3^ platform. The Degree algorithm of Cytoscape software was used to to rank the important genes in PPI networks.

### 2.6. Nomogram model construction

We constructed a nomogram model to predict the risk of neuropathic pain using the “rms” package (Iasonos et al., 2008; Park, 2018). The nomogram model’s performance was evaluated through the calculation of Harrell’s concordance index, which assessed predictive power (Harrell et al., 1996). After that, we used “ROC” package construct the receiver operator characteristic (ROC) curve to validate the diagnostic effectiveness of the candidate biomarkers. We employed the area under the ROC curve (AUC) to indicate the accuracy. A criterion (0.9 ≤ AUC < 1) was used to identify excellent accuracy.

### 2.7. Immune cell analysis

To investigate the function of immune cells in neuropathic pain, we evaluated the level of immune cell infiltration of 22 immunocytes in the neuropathic pain group by CIBERSORT analysis (Chen et al., 2018).

### 2.8. Mendelian randomization

All the data in this study were used in the open database. Two-sample MR was used to explore the causal association between hub gene and the risk of neuropathic pain with the definition of SNPs as IVs. Hub gene data obtained from the publicly available Genome-Wide Association Study (GWAS) data source. We selected the most critical gene, il-2, and trigeminal neuralgia, as representative diseases of neuropathy, for Mendelian randomization analysis. Data on IL-2 is available at https://gwas.mrcieu.ac.uk/datasets/?gwas_id__icontains=prot-a-1512&year__iexact=&trait__icontains=&consortium__icontains= and data on trigeminal neuralgia is available at https://gwas.mrcieu.ac.uk/datasets/?gwas_id__icontains=finn-b-G6_TRINEU&year__iexact=&trait__icontains=&consortium__icontains=. MR analysis was performed based on the “TwoSampleMR” Package, and inverse variance weighting (IVW) was used to assess the relationship between hub gene levels and the risk of neuropathic pain. MR–Egger was used for additional sensitivity analysis (Emdin et al., 2017; Dudbridge, 2021).

## 3. Results

### 3.1. DEGs screening

**Figure 1:**
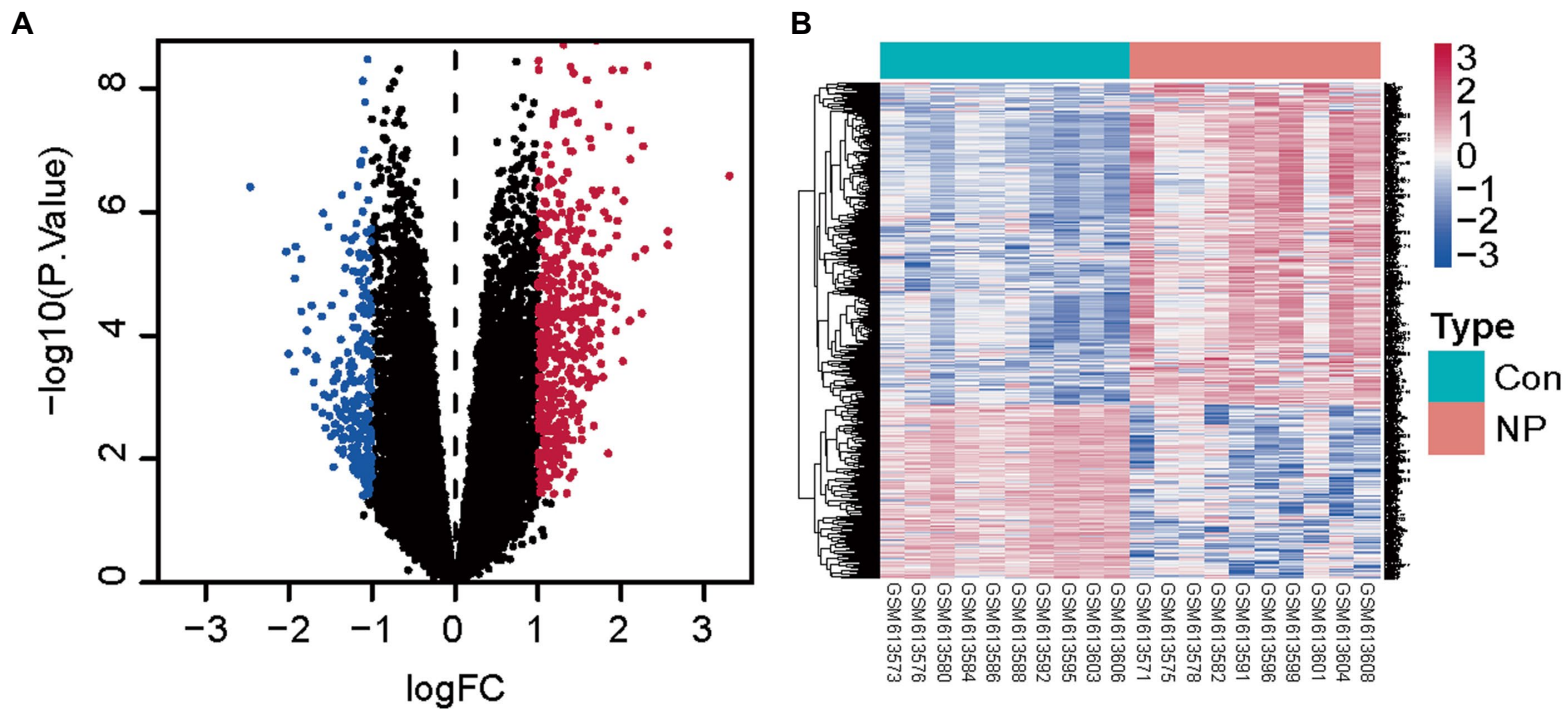
Genes differentially expressed between the neuropathic pain and normal groups. **(A)** Volcanic map for differential expression analysis of GSE24982. **(B)** Heat map for differential expression analysis of GSE24982. Blue represents down-regulated genes, red represents up-regulated genes, and black represents undifferentiated genes.

We first obtained the neuropathic pain dataset (GSE24982) from GEO database and we identified the DEGs of the neuropathic pain dataset. We identified 691 DEGs (449 upregulated and 242 downregulated) in the neuropathic pain group compared to the normal group from the GSE24982 dataset (Figures 1A,B; Supplementary Table S1).

### 3.2. Construction of WGCNA network and identification of neuropathic pain-related module

**Figure 2:**
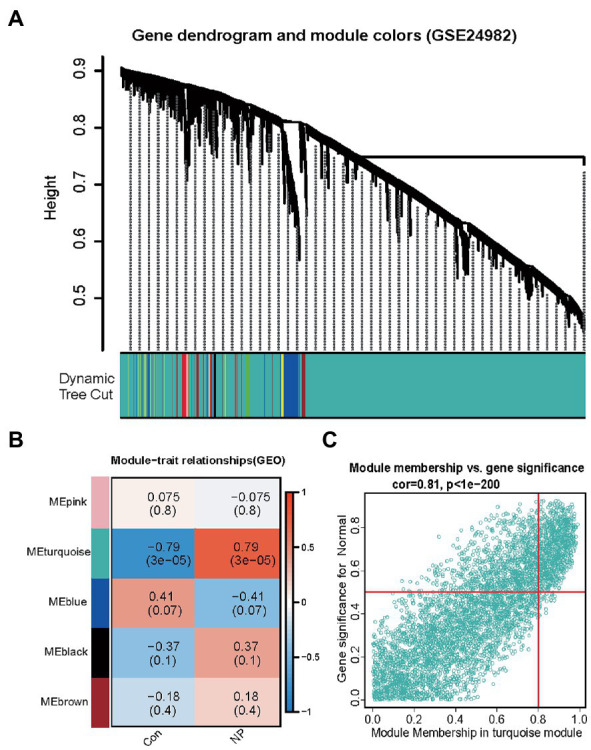
Identification of neuropathic pain-associated gene modules in the GEO dataset using WGCNA. **(A)** Dendrogram of all genes in the GSE24982 dataset was clustered on the basis of a topological overlap matrix (1-TOM). Each branch in the clustering tree represents a gene, while co-expression modules were constructed in different colors. **(B)** Module-trait heatmap of the correlation between the clustering gene module and neuropathic pain in the GSE24982 dataset. Each module contains the corresponding correlation coefficient and *p* value. **(C)** Scatter plot of module cyan has the strongest positive correlation with neuropathic pain in the GSE24982 dataset.

To identify whether or not the potential gene modules were associated with neuropathic pain, we performed WGCNA analysis of all candidate genes from neuropathic pain-related datasets (GSE24982) (Figure 2A). We identified five different modules (Figure 2B). After the analysis of the positive correlation coefficients. At last, module turquoise was screened out in the GSE24982 dataset (Figure 2C; Supplementary Table S2).

### 3.3. Go/KEGG analyses

**Figure 3:**
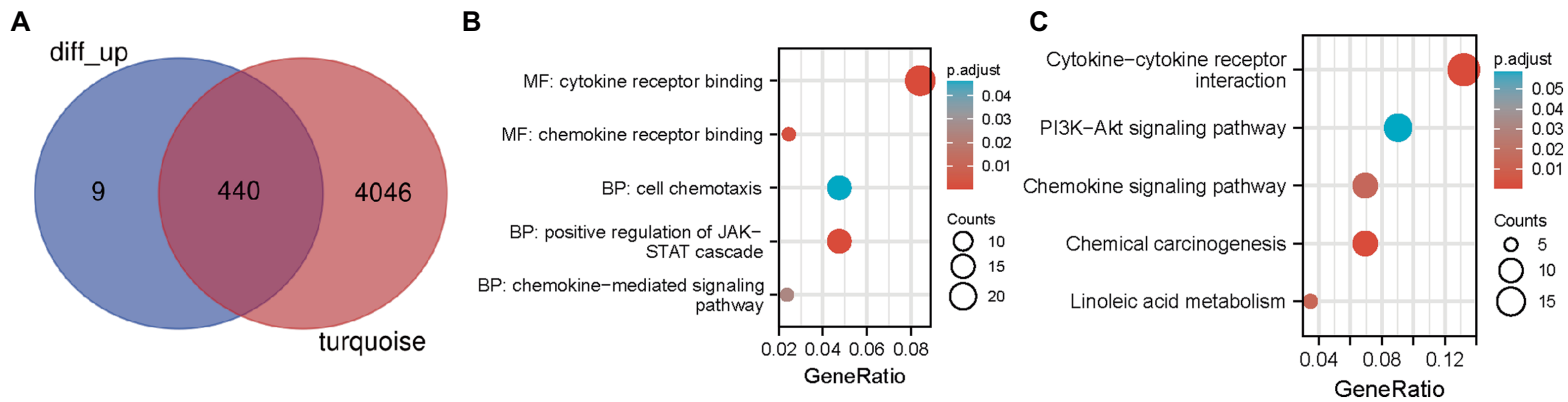
Candidate hub genes were screened and validated. **(A)** Venn diagram revealed 440 overlapping candidate hub genes. **(B)** GO enrichment analysis of candidate hub genes. **(C)** KEGG pathway analysis of candidate hub genes.

To search for co-expressed genes between WGCNA-derived hub genes and DEGs. We eventually screened 440 overlapping genes as candidate hub genes that may play an important role in the development and progression of neuropathic pain (Figure 3A). GO and KEGG analyses were conducted to further explore the underlying roles of these 440 overlapping genes (Figures 3B,C). GO enrichment analysis showed that the overlapping genes mainly affect the biological functions of cytokine receptor binding, chemokine receptor binding, cell chemotaxis and positive regulation of JAK STAT cascade. KEGG enrichment analysis showed that the overlapping genes mainly affect cytokine-cytokine receptor interaction, chemokine-mediated signaling pathway, PI3K AKT pathway, chemokine pathway and chemical carcinogenesis.

### 3.4. PPI network analysis for hub genes

**Figure 4:**
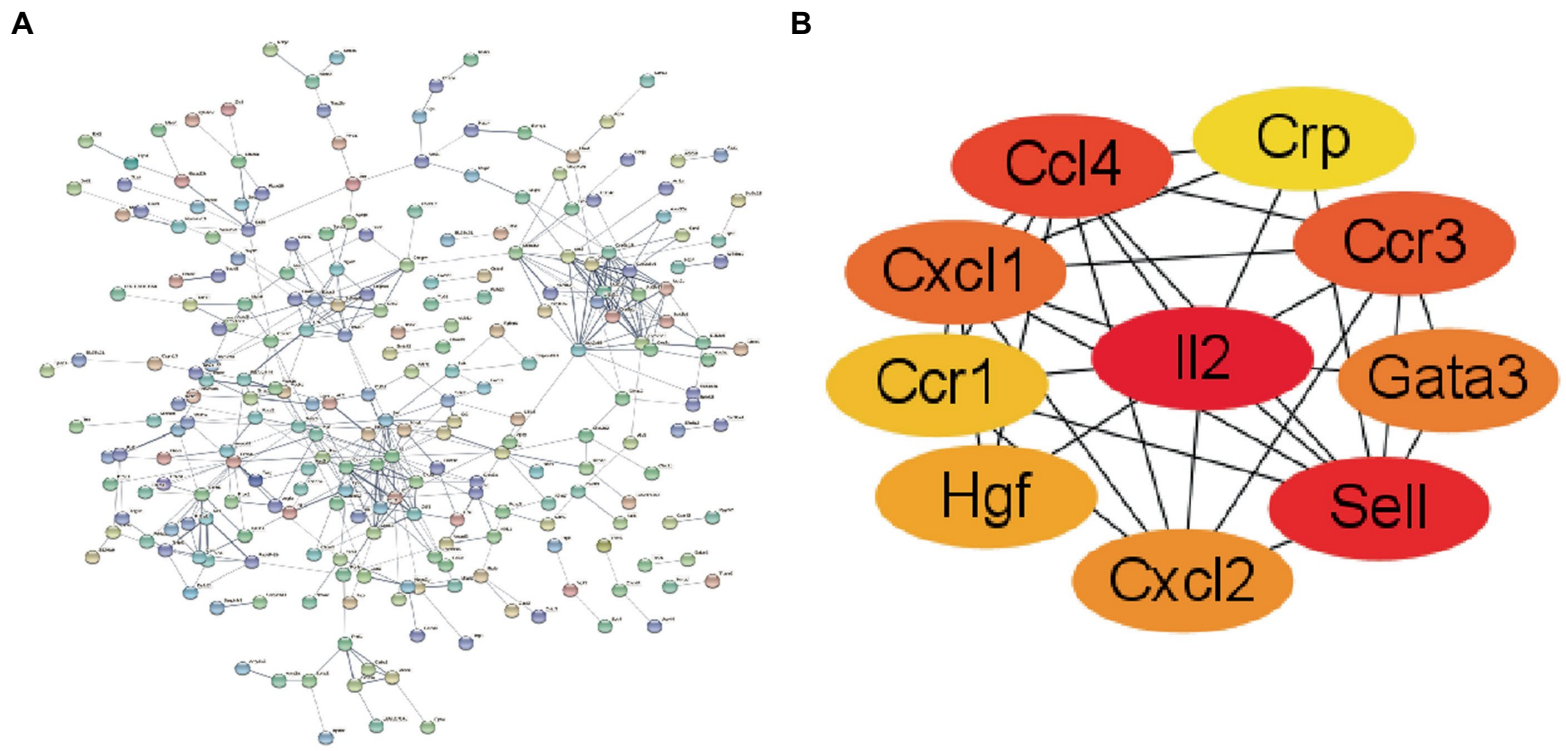
The construction of PPI network. **(A)** PPI network of overlapping hub genes. **(B)** The core genes of the interaction network were obtained by degree ssalgorithm.

We used the STRING online tool to construct a PPI network of overlapping hub genes (Figure 4A). Subsequently, the top ten highly ranked up-regulated genes were then visualized by using Cytoscape software (Figure 4B). Briefly, IL2, SMELL, CCL4, CCR3, CXCL1, CCR1, HGF, CXCL2, GATA3, and CRP were sorted out. The deeper the color, the higher the score.

### 3.5. Construction of nomogram model for neuropathic pain risk prediction

**Figure 5:**
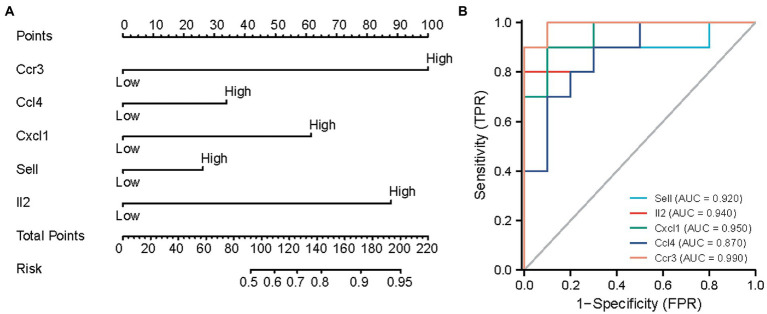
Predicting the risk of neuropathic pain using nomograms. **(A)** Nomogram model of hub genes. **(B)** ROC curves to assess the diagnostic efficacy of nomogram model and each hub gene.

We then constructed a nomogram model to predict neuropathic pain risk (Figure 5A). Thus, our nomogram model performs well in neuropathic pain prediction. Subsequently, we calculated ROC curves for the five hub genes (IL2, SELL, CXCL1, CCL4, and CCR3) to assess the diagnostic effect. The AUC of our nomogram could differentiate between neuropathic pain and controls (Figure 5B). The AUC values of IL2, SELL, CXCL1, CCL4, and CCR3 are, respectively, 0.940, 0.950, 0.870, and 0.990.

### 3.6. Assessment of immune cell infiltration in neuropathic pain

**Figure 6:**
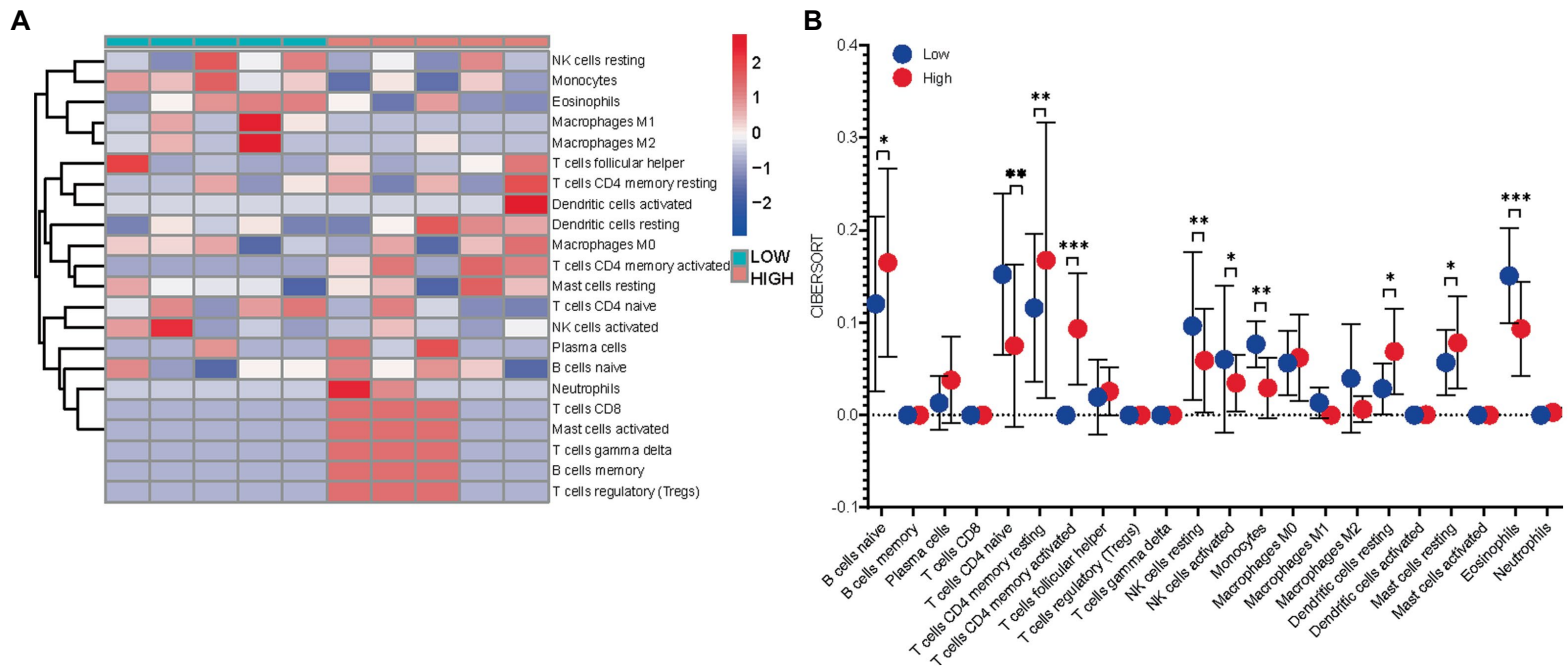
Immuno-correlation of IL2 in neuropathic pain. **(A)** Relative distribution of 22 kinds of immune cells in all neuropathic pain samples. **(B)** The difference of immune cell infiltration between high -and low-expression IL2 groups.

As KEGG pathway analysis and GO enrichment showed that these hub genes were primarily associated with chemokine-mediated pathways. The results shown that the infiltration levels of CD4 resting memory T cells, activated CD4 resting memory T cells, and resting dendritic cells were higher in IL2-high group compared with IL2-low group, while the infiltration levels of activated NK cells, and eosinophiles were lower in IL2-high group than IL2-low groups (Figures 6A,B). These results demonstrate a direct relationship between the immune cell infiltration and hub gene IL2 in neuropathic pain.

### 3.7. IL2 was causally associated with the risk of neuropathic pain

**Figure 7:**
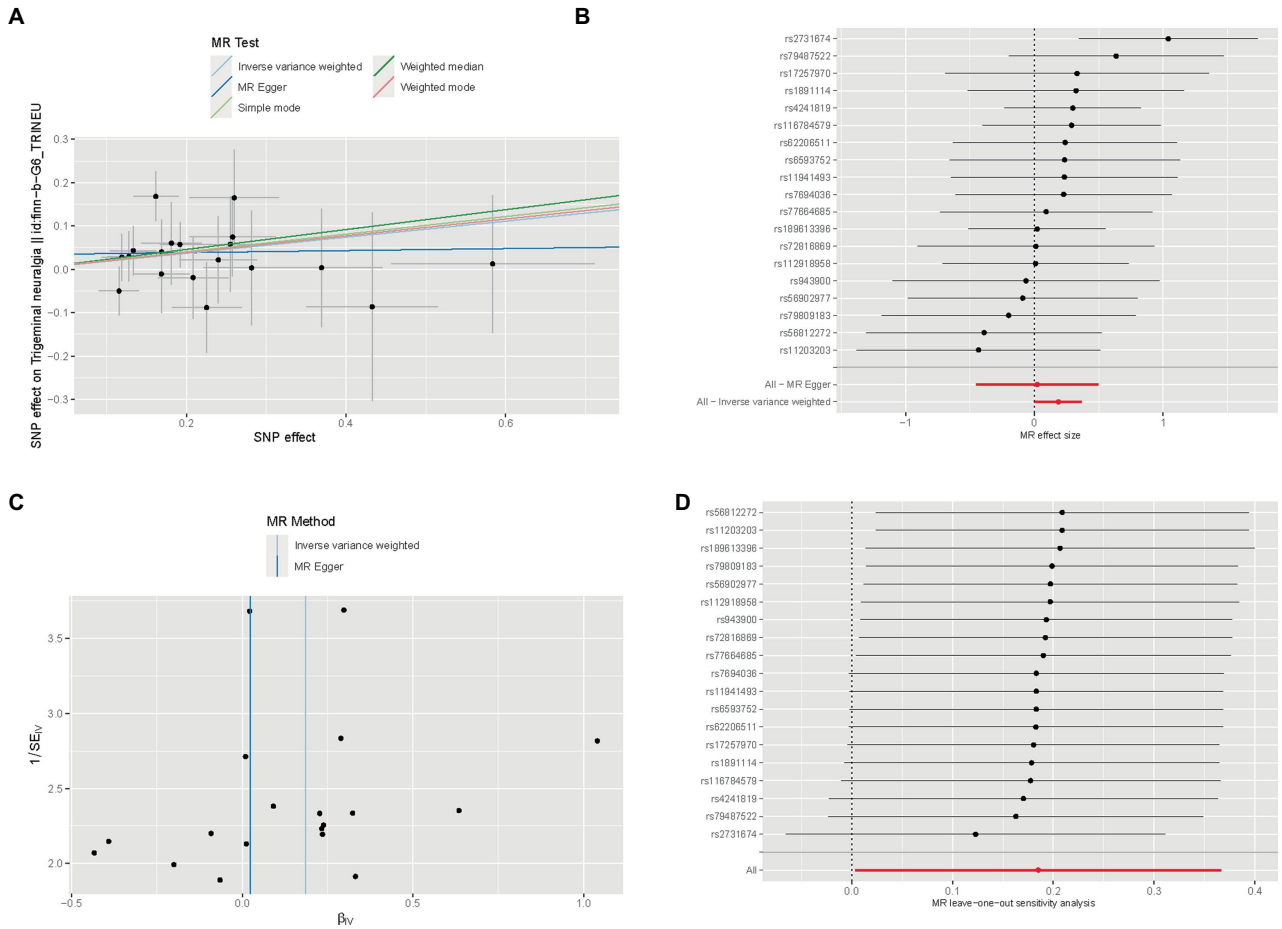
Mendelian randomization study results. **(A)** Scatter plot showing the causal effect of IL2 on the risk of trigeminal neuralgia. **(B)** Forest plot showing the causal effect of each SNP on the risk of trigeminal neuralgia. **(C)** Funnel plots to visualize overall heterogeneity of MR estimates for the effect of IL2 on trigeminal neuralgia. **(D)** Leave-one-out plot to visualize causal effect of IL2 on trigeminal neuralgia risk when leaving one SNP out.

The SNP characteristics of IL2 and trigeminal neuralgia are shown in Supplementary Table S3. All SNPs were not weak instrumental variables. The causal effects of each genetic variation on neuropathic pain are shown in Figures 7A,B. We assessed the causal association between IL2 levels and trigeminal neuralgia. Using the IVW method, we found that IL2 was associated with the risk of trigeminal neuralgia with an OR of 1.203 (95% CI = 1.004–1.443, *p* = 0.045). The MR–Egger method showed no significant statistical significance [OR = 1.023, 95% CI = 0.636–1.647, *p* = 0.925]. The causal effect of the funnel plot was roughly symmetrical (Figures 7C), and the intercept of the MR Egger regression did not observe horizontal pleiotropy (*p* = 0.480), further showing that pleiotropy did not bias the causal effect. As shown in Figure 7D, we systematically performed the MR analysis again on the remaining SNPs after removing each SNP. The results remained consistent, suggesting that the calculation results of all SNPs made causality significant. This also indicates that there was no dominant SNP in IL2 levels and trigeminal neuralgia, and the previous MR results were valid.

## 4. Discussion

Neuropathic pain is a complicated chronic disease that occurs after neurological injury, its precise mechanism remains unclear. Neuralgia is not a single disorder, but a clinical complex presentation caused by a variety of injuries or diseases, and the pathogenesis is diverse and complex (Torta et al., 2017). There are many recent therapeutic approaches targeting the peripheral and central sensitization of neuropathic pain, including NSAIDs, minimally invasive interventions, and ion channel blockers (Binder and Baron, 2016). Nevertheless, these therapies do not offer significant long-term pain alleviation, so alternative contributing mechanisms to neuropathic pain ought to be investigated. With the advancement of molecular biology techniques, the role of pain-associated genes in the progression of neuropathic pain has emerged as an essential research topic (von Hehn et al., 2012). By quantifying the expression levels of thousands of genes in biological specimens concurrently with the genome-wide expression data gained, we are able to study the intricate regulatory associations between genes and potentially find better targets for treatment of neuropathic pain.

Diagnostic biomarkers are designed to identify patients with pathological changes. Currently, researchers are focused on validating biomarkers associated with neuropathic pain. In a recent research, Robust Rank Aggregation (RRA) method has been employed to identify hub genes of neuropathic pain (Cui et al., 2022). Finally, based on the analysis of RRA and PPI analysis, we identified 2 pivotal genes related to neuropathic pain, including Cav1 and Lep. In an earlier study, SCN10A and SST were identified as biomarkers of neuropathic pain by Zhu et al. (2019). In addition, overexpression of the p53 gene in dorsal root ganglion neurons and ensuing increase of caspase-3 expression leads to an increase in apoptosis in these neurons. p53 has also been shown to be partially responsible for chronic constrictive injury-induced neuropathic pain (Gao et al., 2018). SNAP25 plays a role in extracellular release of neurotransmitters and synaptogenesis (Wu and Nie, 2022). In another research, seven genes (Aif1, Atf3, Gfap, Ctss, Scg2, Vgf, and Jun) were identified as the hub genes for neuropathic pain (Xu et al., 2022). However, none of them have validated and assessed the efficacy of candidate biomarkers for diagnosis through mathematical modelling. A more robust and comprehensive design ought to be considered when using these bioinformatics methods to investigate disease. In our research, we first used WGCNA analysis to screen the hub genes of neuropathic pain. We sorted out ten genes, including IL2, SMELL, CCL4, CCR3, CXCL1, CCR1, HGF, CXCL2, GATA3, and CRP as the candidate hub genes for neuropathic pain. Consequently, our nomogram model displayed outstanding performance in the prediction of neuropathic pain. Subsequently, we calculated ROC curves for five hub genes (IL2, SELL, CXCL1, CCL4, and CCR3) to evaluate the diagnostic effect. The AUC of the nomogram can differentiate neuropathic pain from the control group.

There is growing evidence that uninjured DRGs surrounding injured DRGs also act in the onset and progression of neuropathic pain (Fukuoka et al., 2001; He et al., 2014). In this research, we downloaded and analyzed GSE24982 mRNA-seq data and found that 449 genes in the SNL group were up-regulated in comparison to the sham group. Some chemokines (e.g., CCL2, CXCL1, CCL7, CX3CL1, and CCL21) were shown to act on inflammatory and neuropathic pain by mediating neuron-glial interactions in the central nervous system (Imai et al., 2013; Jiang et al., 2016). These results demonstrated once again that chemokines are important in neuropathic pain and may be hub genes influencing the progression of neuropathic pain. The JAK–STAT pathway has been extensively investigated in neurological diseases and has been reported to have a significant role in ischemic stroke (Zhu et al., 2021a,b), glioma (Zhu et al., 2021c), and neuropathic pain (Dominguez et al., 2008; Li et al., 2018). PI3K/AKT pathway also exert crucial role in the progress of neuropathic pain, some agents have analgesic and anti-inflammatory effects on neuropathic pain through inhibition of the PI3K/Akt signaling pathway, such as cryptotanshinone (Zhang et al., 2019), geniposide (Zhang et al., 2022). This confirms that the JAK–STAT and PI3K-AKT pathways are important in neuropathic pain, but whether chemokines and the JAK–STAT or PI3K-AKT pathway are interlinked in neuropathic pain remains to be further investigated.

Chemokines, a small family of secreted proteins, are regulators of peripheral immune cell transport (Charo and Ransohoff, 2006). Chemokines are also expressed in the CNS and modulate CNS function under pathological and physiological conditions, involving synaptic transmission, neuronal development and neuroinflammation related to neurological diseases (Asensio and Campbell, 1999). A study reported that delivery of CXCL1 shRNA lentiviral vectors in the spinal cord before or after SNL consistently diminished SNL-evoked nociceptive responses. CXCL1 application in the spinal cord caused not only nociceptive hyperalgesia but also rapid activation of neurons, as evidenced by the expression of c-Fos and cAMP response element binding protein and phosphorylated extracellular signal-regulated kinase in spinal cord neurons (Asensio and Campbell, 1999). Our study also confirmed that CXCL1 is one of the key genes in neuropathic pain, and therefore inhibiting CXCL1 signaling may provide a novel treatment for neuropathic pain. In addition, we found that CXCL2 is also one of the key genes in neuropathic pain and previous studies showed that CXCL2 levels were elevated on the first day of neuropathic pain induction and gradually decline. Injection of anti-CXCL2 into the trigeminal ganglion reduces pain, suggesting that anti-CXCL2 is a therapeutic option for neuropathic pain (Iwasa et al., 2019). It has also been reported that cc-chemokine ligand 4 (CCL4) is involved in the induction of neuropathic pain after peripheral nerve injury, confirming our results that CCL4 is one of the hub genes. CCR1 was reported to enhance modification of DGCR8 by SUMO1 through phosphorylation of ERK, thereby promoting activation of spinal microglia and inflammatory responses and increasing pain sensitivity in SNL rats (Shi et al., 2022). Our research provides additional potential therapeutic targets for neuropathic pain.

Through using the Cytoscape software, interleukin-2 (IL-2) was ranked the most important gene in the PPI network, as well as the most important gene of the hub gene. IL-2, also called cell growth factor, is an immunomodulatory cytokine excreted by T lymphocytes in response to antigen stimulation (Zhu et al., 2022). Previous studies have revealed the anti-injurious (analgesic) effects of IL-2 in the peripheral and central nervous system (Yao et al., 2002). The short lifespan of IL-2 *in vivo* unfortunately makes clinical application of IL-2 for analgesia impractical. However, a new approach to single intrathecal injections of IL-2 has to be available as a gene therapy for the treatment of neuropathic pain (Yao et al., 2002). The effects of adenovirus-mediated IL-2 genes on chronic neuropathic pain and basal nociceptive response in rats have also been investigated. Yao MZ et al. evaluated the anti-injurious effects of adenovirus type 5 (Ad5) and Ad5-IL2 using radiation heat-induced paw withdrawal latency. Intrathecal injection of Ad5-IL2 was found to have a significant anti-injurious effect on chronic neuropathic pain and basal nociceptive response and was sustained for 4 and 3 weeks, respectively (Yao et al., 2003). This study shows that adenovirus-mediated intrathecal delivery of the IL2 gene has a long-lasting anti-injurious effect. In conclusion, IL2 is a potential therapeutic target for neuropathic pain.

This is the first study to explore the causal association between IL2 levels and trigeminal neuralgia risk by a two-sample MR analysis based on a large amount of GWAS data of IL2 (exposure) and trigeminal neuralgia (outcome). This MR study showed that serum IL2 levels might be causally associated with an increased risk of trigeminal neuralgia. MR is similar in concept to prospective randomized controlled trials (RCTs) but reduces systematic biases that affect the results of traditional observational studies, such as confounding factors and reverse causality. The high accuracy of genotyping can effectively avoid regression dilution caused by detection errors. To ensure that SNPs are not related to any confounding factors between IL2 and trigeminal neuralgia, we chose only participants from European populations. Finally, to ensure the stability of the results, we also performed MR–Egger regression test, and no evidence of directional level pleiotropy was observed.

Despite these meaningful findings, our research still has a few shortcomings. Firstly, this study only used one dataset because of the absence of microarray data in the area of neuropathic pain, and the results would have been more convincing if more neuropathic pain-related datasets had been combined. Secondly, this study only used bioinformatics to analyze the hub genes and potential functions associated with the occurrence of neuropathic pain, and more biological experiments are required to validate the specific mechanisms of the hub genes screened.

## 5. Conclusion

We constructed a WGCNA-based co-expression network and identified neuropathic pain-related hub genes. This may help in the development of pre-symptomatic diagnostics and may provide further insights into the molecular mechanisms for understanding neuropathic pain risk genes.

## Data availability statement

The original contributions presented in the study are included in the article/Supplementary material, further inquiries can be directed to the corresponding authors.

## Author contributions

LL and JL designed the study and revised the manuscript. JZ and CL acquired the raw data and analyzed the data. JZ wrote the manuscript. All authors contributed to the article and approved the submitted version.

## Conflict of interest

The authors declare that the research was conducted in the absence of any commercial or financial relationships that could be construed as a potential conflict of interest.

## Publisher’s note

All claims expressed in this article are solely those of the authors and do not necessarily represent those of their affiliated organizations, or those of the publisher, the editors and the reviewers. Any product that may be evaluated in this article, or claim that may be made by its manufacturer, is not guaranteed or endorsed by the publisher.

## Footnotes

^1^ https://www.ncbi.nlm.nih.gov/geo/query/acc.cgi?acc=GSE24982

^2^ https://string-db.org/

^3^ https://cytoscape.org/
